# Exercise modality influences lactate production and RPE: running vs. cycling, intervals vs. continuous

**DOI:** 10.3389/fphys.2026.1822989

**Published:** 2026-06-11

**Authors:** Cheng Zhang, Zhenhe Dong, Ran Feng, Linxiao Wu, Jianfeng Li, Yan Zhang

**Affiliations:** 1 Sports & Medicine Integration Research Center (SMIRC), Capital University of Physical Education and Sports, Beijing, China; 2 Taizhou Vocational College of Science & Technology, Taizhou, Zhejiang, China; 3 Yangfangdian Hospital, Beijing, China; 4 School of Physical Education, Shandong University of Technology, Shandong, China; 5 Affiliated Rehabilitation Hospital of Capital University of Physical Education and Sports, Beijing, China; 6 Emerging Interdisciplinary Platform for Medicine and Engineering in Sports (EIPMES), Beijing, China

**Keywords:** exercise modality, exercise prescription, lactate accumulation, rating of perceived exertion (RPE), sex differences

## Abstract

**Introduction:**

Lactate has been redefined from a metabolic waste product to a key signaling molecule regulating energy metabolism, gene expression, and disease progression, making the precise regulation of exercise-induced lactate exposure critical for population-specific prescriptions (e.g., for the elderly or cancer patients). The aim of this study was to investigate the coupling between lactate accumulation and subjective fatigue by comparing whole-body (running) versus localized (cycling) exercise, as well as intermittent versus continuous modalities.

**Methods:**

Twelve healthy adults (six males and six females) participated in this study, completing combinations of three intensities and two modalities: Moderate-Intensity Interval Training (MIIT), Moderate-Intensity Continuous Training (MICT), and High-Intensity Interval Training (HIIT) via running and cycling ergometry. Blood lactate area-under-curve (AUC) was calculated, and Lactate Production Efficiency (LPE = AUC/RPE) was innovatively proposed to quantify lactate exposure per unit of Rating of Perceived Exertion (RPE).

**Results:**

Cycling induced 59% (MIIT, p < 0.01) and 67% (HIIT, p < 0.05) higher lactate AUC than running, irrespective of intensity or intermittency. Furthermore, HIIT cycling yielded a 52% higher LPE than running (20.48 vs. 13.47 mmol·min/L·scale, p < 0.01), indicating superior lactate stress per fatigue unit and optimizing lactate elevation, whereas running MIIT minimized lactate exposure. Males showed 36–43% higher running AUC than females (p < 0.05), suggesting heightened metabolic sensitivity. Interval efficacy was also confirmed, as HIIT increased lactate AUC by 44–87% versus MICT (p < 0.05).

**Discussion:**

The introduction of LPE effectively quantifies exercise-modality effects on fatigue-lactate decoupling. The findings demonstrate metabolic stress concentration in localized exercise (cycling) and male-specific lactate sensitivity during whole-body running, providing RPE-based strategies for precision exercise prescription and advancing personalized interventions in sports medicine.

## Introduction

For decades, lactate produced during exercise was predominantly viewed as the end-product of glycolysis and a marker of muscular fatigue, its role in the human body simplistically reduced to that of a “metabolic waste product” (Sun et al., 2017). However, with the advent of the “lactate shuttle” theory (Brooks, 2018) and the discovery of “histone lactylation” mechanisms (Zhang et al., 2019), the physiological functions of lactate have been fundamentally redefined. Lactate is no longer considered merely a metabolic by-product; instead, it has emerged as a key signaling molecule playing multifaceted roles in energy metabolism, gene regulation, and cellular signaling (Kanova and Kohout, 2022; Qin et al., 2025).

In the context of health promotion, the beneficial effects of lactate are gaining increasing recognition (Li et al., 2022). As a cross-tissue energy substrate, lactate can be transported via the “lactate shuttle” to organs such as the liver, heart, and brain, supporting their function (Suzuki et al., 2011). For instance, lactate promotes skeletal muscle mitochondrial biogenesis (Hoshino et al., 2015), mediates myocyte proliferation and differentiation (Ohno et al., 2018), and serves as a significant energy source for the brain, enhancing cognitive function (Dienel, 2014; Jimenez-Maldonado et al., 2018) Studies indicate that lactate can augment lactylation levels at the K1897 site of myosin α-MHC, counteracting myocardial injury and aiding in heart failure treatment (Mao et al., 2024). Furthermore, lactylation modification has been shown to reduce appetite, contributing to weight management (Li et al., 2022). Consequently, elevating lactate levels through exercise may represent a viable strategy for optimizing health in populations seeking metabolic enhancement, muscle function preservation, brain health improvement, cardiac rehabilitation, or weight loss.

Nevertheless, the physiological role of lactate exhibits significant duality. While beneficial for metabolic and muscular adaptations, excessive lactate accumulation can pose risks under certain pathological conditions, such as exacerbating the tumor microenvironment or potentially suppressing immune responses (Marchiq and Pouysségur, 2016; Zhang and Li, 2020; Chen et al., 2024). Consequently, distinct populations exhibit divergent requirements for exercise-induced lactate: some individuals necessitate efficient elevation of lactate through exercise to maximize metabolic benefits, whereas others (e.g., cancer patients or the elderly) require precise control of exercise intensity to avoid excessive metabolic stress (Zhou et al., 2025; Grasa et al., 2023; Halford et al., 2023). This paradoxical nature underscores the critical importance of precisely quantifying and regulating total lactate exposure during personalized exercise rehabilitation.

Traditionally, exercise prescriptions heavily rely on subjective fatigue metrics, such as the Rating of Perceived Exertion (RPE), to monitor intensity. However, a fundamental mismatch often exists between subjective perception and actual physiological stress, suggesting that different exercise modalities may decouple RPE from blood lactate accumulation. Furthermore, isolated blood lactate measurements only capture a transient snapshot of the dynamic balance between lactate production and clearance. To more accurately quantify total metabolic stress throughout an exercise session, the Area Under the Curve (AUC) of blood lactate offers a robust alternative. To further elucidate this fatigue-lactate decoupling, we propose a novel metric: Lactate Production Efficiency (LPE), defined as the lactate AUC per unit of RPE. By utilizing AUC and LPE, this study aims to evaluate how different exercise modalities influence this decoupling, ultimately providing a quantifiable basis for designing precision exercise prescriptions that optimize the therapeutic window of lactate.

Exercise physiology mechanisms indicate that lactate production is closely linked to muscle recruitment patterns and energy metabolism pathways (Jensen-Urstad et al., 1994). Exercises predominantly engaging localized large muscle groups (e.g., cycling ergometry) often impose concentrated load per muscle unit, predisposing to local hypoxia and activating the glycolytic pathway, potentially yielding higher lactate levels. Conversely, exercises involving multiple muscle groups across the whole body (e.g., running) may exhibit relatively lower lactate production efficiency due to dispersed energy expenditure. Furthermore, the periodic superimposition of high-intensity bouts during intermittent exercise may exacerbate anaerobic metabolism, theoretically inducing greater lactate accumulation compared to continuous exercise(Rodriguez et al., 2018). However, the quantitative relationship between lactate production and subjective fatigue perception (Rating of Perceived Exertion, RPE) across different exercise modalities (localized vs. whole-body, intermittent vs. continuous) remains inadequately characterized, limiting the precision of exercise prescription design (Green et al, 2004).

To address this gap, the present study systematically compared lactate production and RPE responses by employing two representative exercise modalities—treadmill running (whole-body, multi-muscle group exercise) and cycling ergometry (localized large lower-limb muscle group exercise)—across three distinct protocols: Moderate-Intensity Continuous Training (MICT), Moderate-Intensity Interval Training (MIIT), and High-Intensity Interval Training (HIIT). Central to our analysis, we introduce the novel metric Lactate Production Efficiency (LPE), calculated as the lactate AUC per unit of RPE. Rather than merely quantifying which modality generates more lactate, this study seeks to identify exercise strategies that achieve maximum lactate-driven health benefits with minimum subjective fatigue. Such a “high-efficiency” approach is particularly critical for clinical populations—such as the elderly, cancer survivors, or those in cardiac rehabilitation—who require potent metabolic and signaling stimuli but possess limited tolerance for high-intensity physical exertion. By elucidating how exercise modality decouples physiological stress from subjective perception, these findings aim to provide a robust, quantifiable basis for designing personalized, precision exercise prescriptions that optimize the therapeutic window of lactate.

## Materials and methods

### Study design

This study employed a randomized controlled crossover design.Participants were recruited based on the following criteria: (1) healthy adults aged 18–30 years; (2) no history of cardiovascular, respiratory, or metabolic diseases; (3) no musculoskeletal injuries in the past 6 months. Exclusion criteria included: (1) smoking; (2) taking medication that affects heart rate or metabolism.Ethical approval was granted by the Capital University of Physical Education and Sports Ethical Committee.(Approval Review Number:2024A023)This study followed CONSORT guidelines for a randomized controlled trial, and informed consent has been obtained from all subjects.

**Figure 1:**
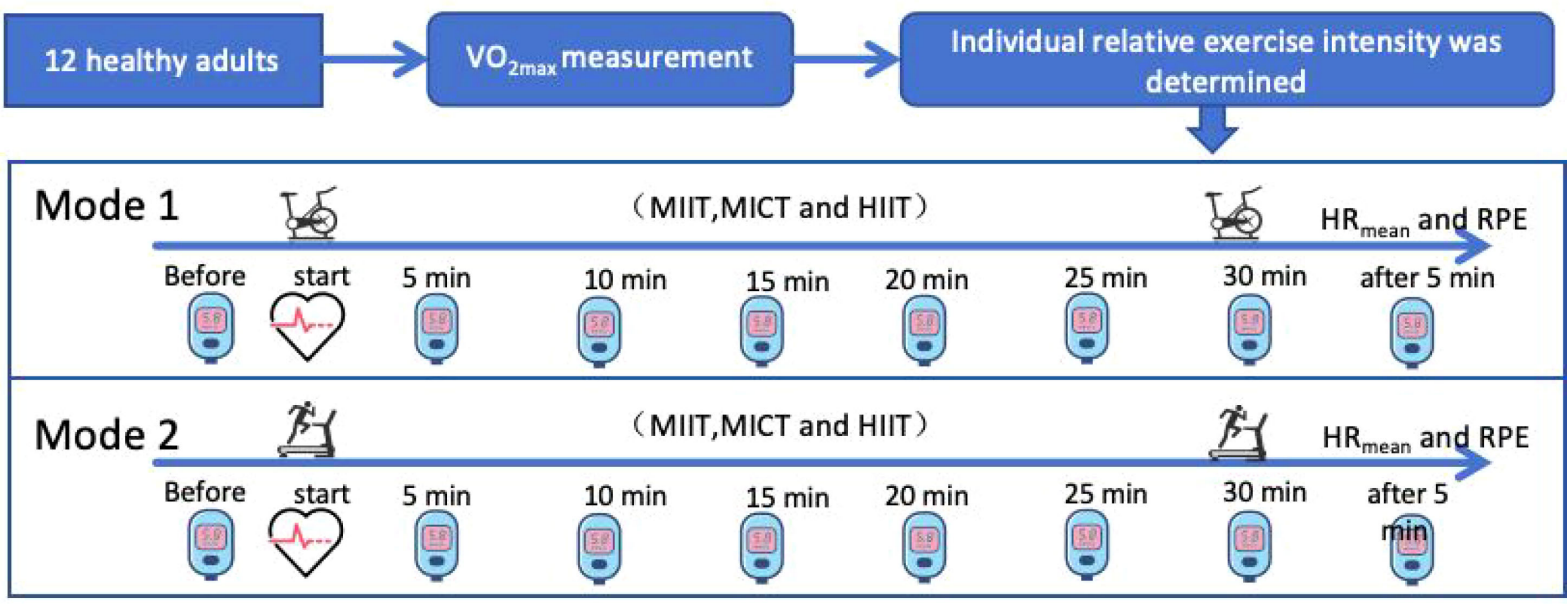
Schematic representation of the experimental design. The study employed a randomized cross-over design involving 12 healthy participants (6 males and 6 females). All participants underwent six distinct exercise trials: three exercise protocols (MICT, MIIT, and HIIT) across two modalities (running and cycling ergometry). **(A)** Overview of the trial sequence and 48-hour washout period between sessions. **(B)** Specific parameters for each protocol: MICT (continuous exercise at 40-59%HRR), MIIT (4-min bouts at 40-59%HRR with 1-min rest), and HIIT (4-min bouts at 60-89%HRR with 1-min rest). **(C)** Timeline for blood lactate sampling and RPE assessment, conducted at baseline, during exercise, and throughout the 15-minute recovery phase.

Prior to the experiment, all participants underwent cardiopulmonary exercise testing (CPET) to determine their maximal oxygen uptake (VO_2max_), which served as the basis for setting subsequent exercise intensities (Laveneziana et al., 2021) (see **Table 1**). Participants were required to complete three exercise protocols: MIIT, MICT, and HIIT. MICT consisted of 30 minutes of continuous exercise at a target intensity of 40-59%HRR or 46-63%VO2max. MIIT comprised 6 sets 4-minutes exercise bouts at target intensity of 40-59%HRR or 46-63%VO2max,interspersed with 1-minute rest periods. To ensure method consistency and to eliminate the possible influence of posture on the degree of perceived exertion, rest was performed in the sitting position for both exercise modes. HIIT consisted of 6 sets of 4-minute exercise bouts at a target intensity of 60-89%HRR or 64-90%VO2max,interspersed with 1-minute same rest method. Each protocol was performed under two exercise modalities: cycling ergometry and treadmill running. Each experimental session was separated by a washout period of at least 48 hours to minimize fatigue and carry-over effects (see **Figure 1** and **Table 2**).

**Table 1:** Basic physical data of participants in the experiment.

Variable	Male(n=6)	Female(n=6)	P	η^2^
Age(yr)	27.2 ± 5.8	23.3 ± 2.8	0.214	0.15
Height(cm)	179.8 ± 7.3	163.5 ± 5.3	0.002^*^	0.62
Weight(kg)	80.3 ± 9	53.3 ± 5.6	<0.001^*^	0.765
BMI(kg·m-2)	24.4 ± 1.6	19.7 ± 1.3	<0.001^*^	0.716
VO2max(ml·kg-1·min-1)	48.1 ± 5.2	43.2 ± 4.4	0.134	0.21

^*^Significantly different between the groups. BMI, body mass index; VO_2_max, maximal oxygen uptake.

**Table 2:** Experimental condition settings.

Form of Test	Intensity (HRR)	Intensity (VO2max)	Duration (min)	Rest time (min)	Set	Rest type
MIIT	40-59%	46-63%	4	1min	6	Negative
MICT	40-59%	46-63%	30	NO	1	NO
HIIT	60-89%	64-90%	4	1min	6	Negative

The experimental procedure consisted of a warm-up, the main exercise phase, and post-exercise measurements. The warm-up phase included 5 minutes of dynamic stretching followed by 3 minutes of cycling at 50W, with a 1-minute rest before commencing the main exercise. During exercise, capillary blood samples were collected from the fingertip every 5 minutes for blood lactate measurement. Heart rate (HR) was monitored in real-time using a Polar HR monitor (H10, China) to ensure exercise intensity compliance. Immediately post-exercise, participants’ average HR and RPE were recorded. Specific exercise intensities and durations are detailed in **Table 2**.

### Cardiopulmonary exercise testing

Cardiopulmonary exercise testing (CPET) was performed on a treadmill using the standardized BRUCE protocol. This protocol was selected because its progressive increments in both speed and incline safely and effectively elicit true systemic VO_2max_ in healthy, non-athletic adults within the optimal 8–12 minute window. This approach prevents premature test termination due to localized muscular fatigue or biomechanical limitations associated with high-speed flat running. Respiratory gas exchange was continuously measured using a breath-by-breath method via a metabolic cart (Ganshorn, Germany). To minimize artifact noise and breathing irregularities, the raw breath-by-breath data were smoothed and averaged over 10 second intervals. The VO_2max_ was defined as the highest averaged VO_2_ value recorded over consecutive intervals meeting the achievement criteria.

### Heart rate monitoring

Heart rate (HR) was recorded continuously throughout the exercise and active recovery periods using a Polar heart rate monitor (H10, China). To more accurately prescribe and standardize exercise intensity across participants, Heart Rate Reserve (HRR) was utilized instead of maximal heart rate.

### Blood lactate measurement

Baseline blood lactate samples were collected 10 minutes before each exercise session. Capillary blood samples were obtained from the fingertip at minutes 5, 10, 15, 20, 25, and 30 during the exercise phase, and additionally at minute 5 post-exercise. Blood lactate concentration was measured immediately using a portable lactate analyzer (EKF Biosen, Lactate Scout 4, Germany).

### Area under the blood lactate-time curve analysis

The Area Under the Blood Lactate-Time Curve (AUC) served as the key metric for quantifying the total lactate exposure throughout the exercise session. By integrating changes in blood lactate concentration over the entire exercise duration, AUC overcomes the limitation of single time-point measurements and facilitates comparison of the overall lactate accumulation burden across different exercise modalities. AUC was calculated using the trapezoidal method according to the following formula:


$$AUC=\sum_{i=1}^{n-1}\;\frac{\left(C_{i}+C_{i+1}\right)}{2}\times (t_{i+1}-t_{i})$$

where C_i_ represents the blood lactate concentration (mmol/L) at the *i*-th time point, and t_i_ represents the sampling time (min). This method has been validated as an effective means of quantifying exercise metabolic load and is particularly suitable for comparing lactate accumulation profiles across different exercise modalities. Compared to single time-point measurements, AUC provides a more comprehensive characterization of the metabolic response to exercise. For instance, Valborg Land et al. demonstrated that lactate AUC is twice as sensitive as peak VO_2_(VO_2_peak) in detecting the effects of exercise interventions and effectively differentiates the efficacy between distinct exercise types (Valborgland et al., 2021).

### RPE measurement

The RPE was assessed using the Borg RPE scale (Liguori and American College of Sports Medicine, 2020). Participants were asked to report their RPE value immediately following the completion of each exercise session.

### Lactate production efficiency

To quantify the relationship between objective physiological stress and subjective fatigue across different exercise modalities, we introduced the Lactate Production Efficiency (LPE) metric. LPE represents the systemic lactate exposure generated per unit of perceived exertion and was calculated as the ratio of the total lactate area under the curve (Lactate_AUC_) to the Rating of Perceived Exertion (RPE) recorded during the exercise session:


$$LPE=\frac{\text{LactateAUC}}{RPE}$$

where Lactate_AUC_ is expressed in mmol·min/L and RPE is the average of RPE scores collected at the end of exercise. A higher LPE value indicates a greater metabolic efficiency, where a specific level of subjective fatigue is associated with a higher systemic lactate stimulus, a decoupling effect particularly relevant for clinical exercise prescription.

### Statistical analysis

Statistical analyses were performed using Python software (Version 3.12.1, Python Software Foundation, Netherlands), and data visualizations (bar graphs, box plots, and raincloud plots) were generated using GraphPad Prism (Version 10.4.1, GraphPad Software, USA). Continuous data were assessed for normality using the Shapiro-Wilk test. Normally distributed data are presented as mean ± standard deviation (SD), whereas non-normally distributed data are expressed as median and interquartile range (IQR).Based on the study’s hypotheses and crossover design, statistical tests were applied as follows:

Within-participant comparisons: Differences across various exercise conditions (e.g., running vs. cycling; MIIT vs. MICT vs. HIIT) were analyzed using paired t-tests for normally distributed data. For non-normally distributed data, Wilcoxon signed-rank tests were employed. Specifically, all calculated AUC values were confirmed to be normally distributed and were thus analyzed using paired t-tests.

Between-sex comparisons: Differences between sexes (male vs. female) within the same exercise condition were evaluated using independent samples t-tests.

Although an *a priori* sample size calculation was not performed, a *post hoc* power analysis was conducted based on the primary outcome (Lactate AUC) to ensure the reliability of the results. Utilizing a crossover design—which inherently reduces inter-subject variability—the study demonstrated a large effect size (Cohen’s d > 1.2) for the primary comparisons (e.g., cycling vs. running). Consequently, the calculated statistical power (1-β) for detecting the observed differences exceeded 0.90. This indicates that the sample size of 12 participants was highly sufficient to support the robust conclusions of this study despite the relatively small cohort.Statistical significance for all tests was set at P < 0.05.

## Results

### Significantly higher lactate accumulation in localized large-muscle group exercise (cycling) compared to whole-body exercise (running)

Post-experiment analysis confirmed that the average HR for moderate-intensity exercise protocols (MICT and MIIT) was maintained between 40–59% HRR. For the high-intensity exercise protocol (HIIT), the average HR was maintained between 60–89% HRR (see **Table 2**). To achieve the targeted relative intensities, the absolute workloads varied across modalities. During the HIIT protocols, the average running speed was 8.91 ± 0.76 km/h, and the average cycling power output was 162.5 ± 23.6 Watts. For the MIIT, the average running speed was 7.24 ± 0.6 km/h, and the average cycling power was 127.5 ± 17.9Watts. For the MICT protocols,the average running speed was 6.92 ± 0.68 km/h, and the average cycling power was 119.2 ± 16.3Watts (**Table 3**).

**Table 3:** Heart rate (HR), rate of perceived exertion (RPE) and exercise performance during moderate and high-intensity interval training on cycling and running.

Variable	Cycling (MIIT)	Running (MIIT)	Cycling (HIIT)	Running (HIIT)	Cycling (MIIT)	Running (MIIT)
HR, bpm	132.5 ± 4.8	135.2 ± 6.83	155.6 ± 9.82	158.8 ± 6.39	136.5 ± 3.04	138.4 ± 3.29
RPE,6-20 scale	12.5 ± 1.89	12.58 ± 1.66	16.17 ± 1.07	14.58 ± 1.75	12.8 ± 1.47	12.8 ± 1.74
Speed or Power	127.5 ± 17.9watts	7.24 ± 0.6 km/h	162.5 ± 23.6watts	8.91 ± 0.74 km/h	119.2 ± 16.3watts	6.92 ± 0.68 km/h

Average Running Speed(km/h);Average Cycling Power(Watts).

**Figure 2:**
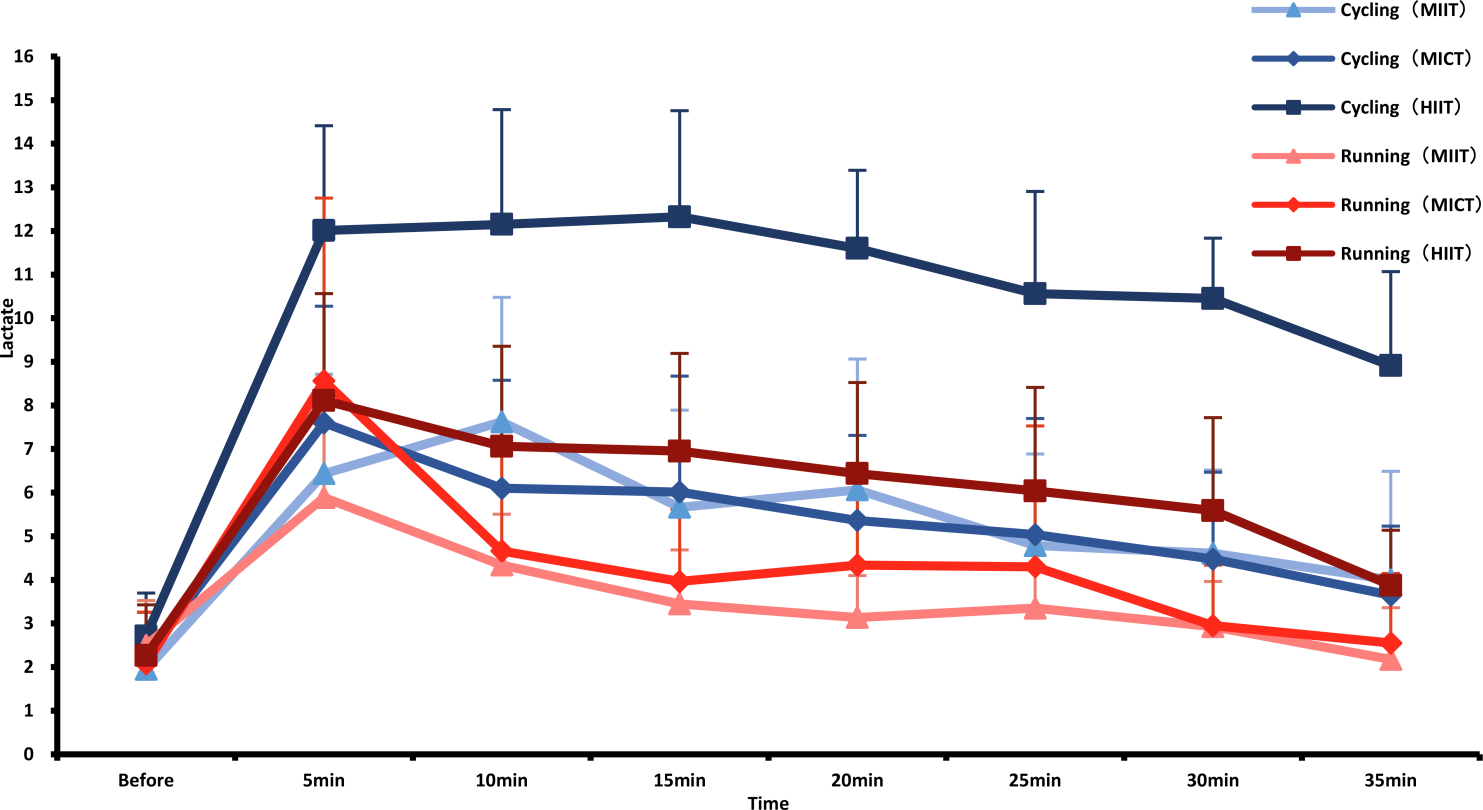
Blood lactate concentration over time across six exercise modalities. Values are presented as mean ± standard deviation (SD). The x-axis represents measurement time points (Before exercise, 5, 10, 15, 20, 25, 30 minutes during exercise and 5 minutes into the recovery period), and the y-axis represents blood lactate concentration (mmol/L).

**Figure 2** illustrates the time-course of blood lactate kinetics across the six exercise modalities. While Cycling HIIT (C-HIIT) clearly elicited the highest and most distinct lactate response, the trajectories of the other modalities (R-HIIT, C-MIIT, R-MIIT, C-MICT, and R-MICT) exhibited substantial temporal overlap and fluctuation. Due to the dynamic balance of lactate appearance and clearance, especially during the work-to-rest transitions in interval protocols, discrete time-point comparisons are insufficient to differentiate the overall physiological stress. These visual ambiguities highlight the limitation of relying solely on single time-point snapshots. Therefore, to accurately capture and quantify the cumulative systemic metabolic burden, we calculated the Area Under the Curve (AUC) for each protocol.

**Figure 3:**
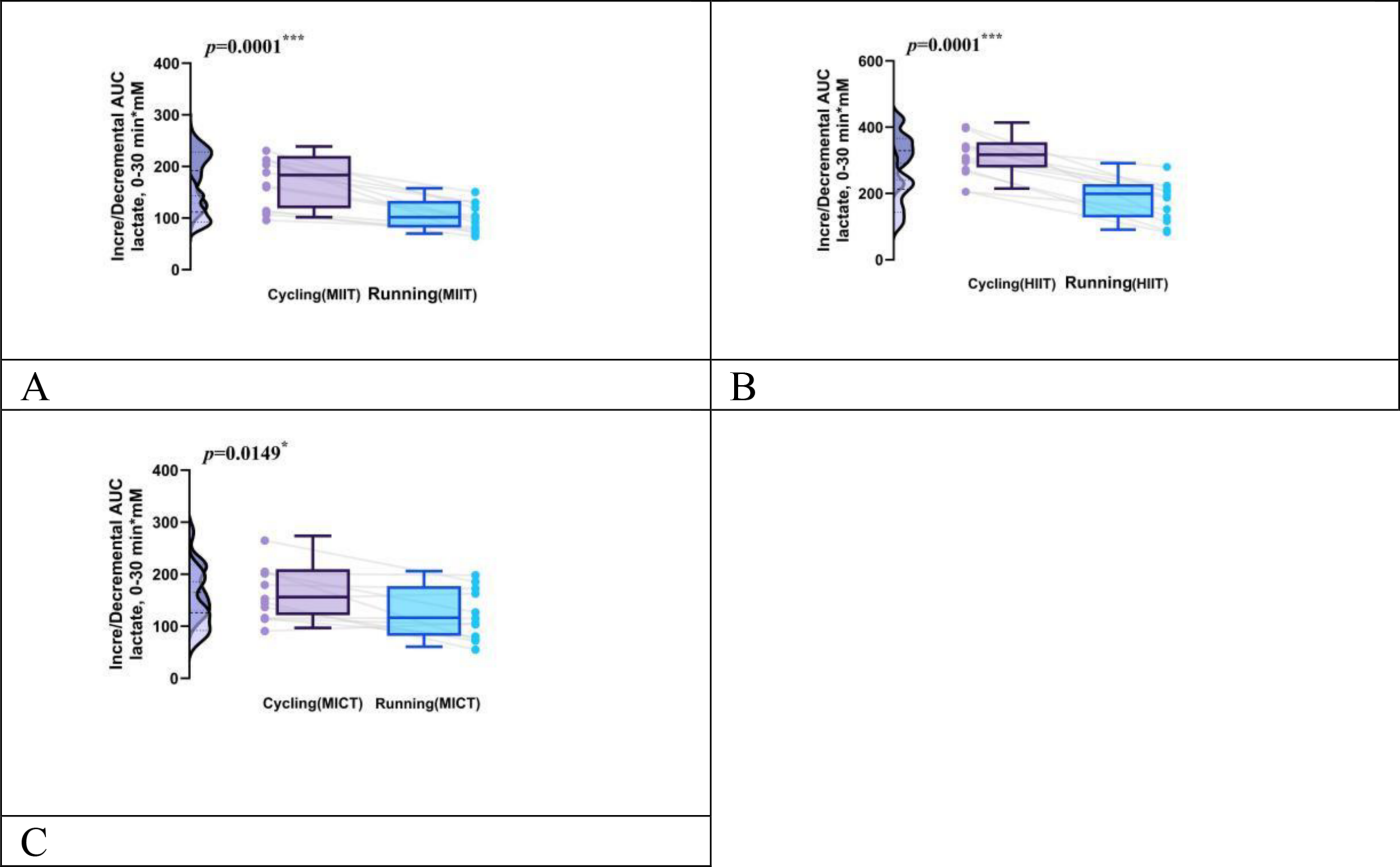
Comparison of blood lactate concentrations between cycling and Running exercises. Data are presented as mean ± SD (n =12). **(A)** Dynamic changes in blood lactate levels during MIIT for both exercise modalities. **(B)** Dynamic changes in blood lactate levels during HIIT. **(C)** Dynamic changes in blood lactate levels during MICT. Cycling induced significantly higher peak lactate and overall accumulation compared to running at equivalent relative intensities.AUC was measured in mmol/L·min. Statistical significance between modalities at specific time points is indicated by asterisks (*p< 0.05,**p< 0.01,***p < 0.001).

Analysis of the AUC revealed that lactate accumulation was significantly higher during cycling ergometry compared to running, regardless of intensity (moderate: MIIT/MICT or high: HIIT) (**Figure 3**): During MIIT the lactate AUC for cycling (183.19 ± 47.05 mmol·min/L) was 59% higher than for running (115.19 ± 26.50 mmol·min/L) (P = 0.000098, t=6.085). During HIIT the lactate AUC for cycling (329.33 ± 60.38 mmol·min/L) was 67% higher than for running (197.29 ± 57.85 mmol·min/L) (P = 0.000019,t=7.406).

### No significant difference in lactate accumulation or subjective fatigue between intermittent and continuous exercise at moderate intensity

**Figure 4:**
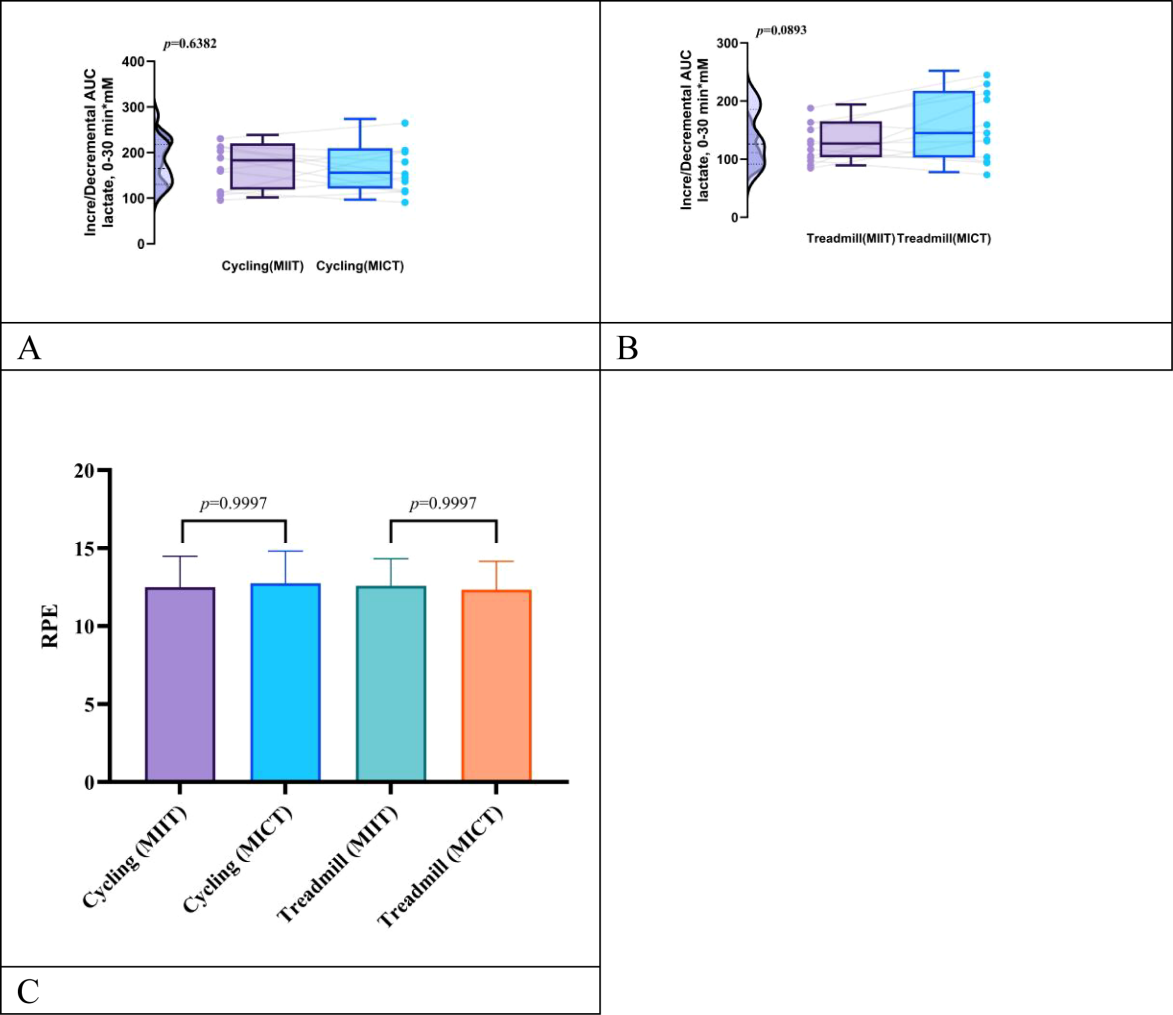
Comparison of blood lactate concentrations and perceived exertion (RPE) between MICT and MIIT. Data are presented as mean ± SD (n = 12). **(A)** Time-course of blood lactate levels during continuous vs. interval cycling. **(B)** Time-course of blood lactate levels during continuous vs. interval running. Note that despite the 1-minute rest periods in MIIT, total lactate accumulation (AUC) did not differ significantly from MICT at the same relative intensity. **(C)** Changes in Rating of Perceived Exertion (RPE) across the four exercise modes.AUC was measured in mmol/L·min. No significant difference in subjective fatigue was observed between MICT and MIIT within the same modality.

Comparison between MIIT and MICT showed:

- Lactate Accumulation: No significant difference was observed between cycling MIIT (183.19 ± 47.05 mmol·min/L) and cycling MICT (175.90 ± 48.84 mmol·min/L) (P = 0.638,t=0.483). Similarly, no significant difference existed between running MIIT (115.19 ± 26.50 mmol·min/L) and running MICT (136.58 ± 46.25 mmol·min/L) (P = 0.089,t=-1.864; **Figures 4A, B**).
- Subjective Fatigue (RPE): No significant differences in RPE values were found between the intermittent and continuous protocols for either exercise modality (P = 0.9997; **Figure 4C**).

### Highest lactate production efficiency in intermittent cycling, lowest in intermittent running

**Figure 5:**
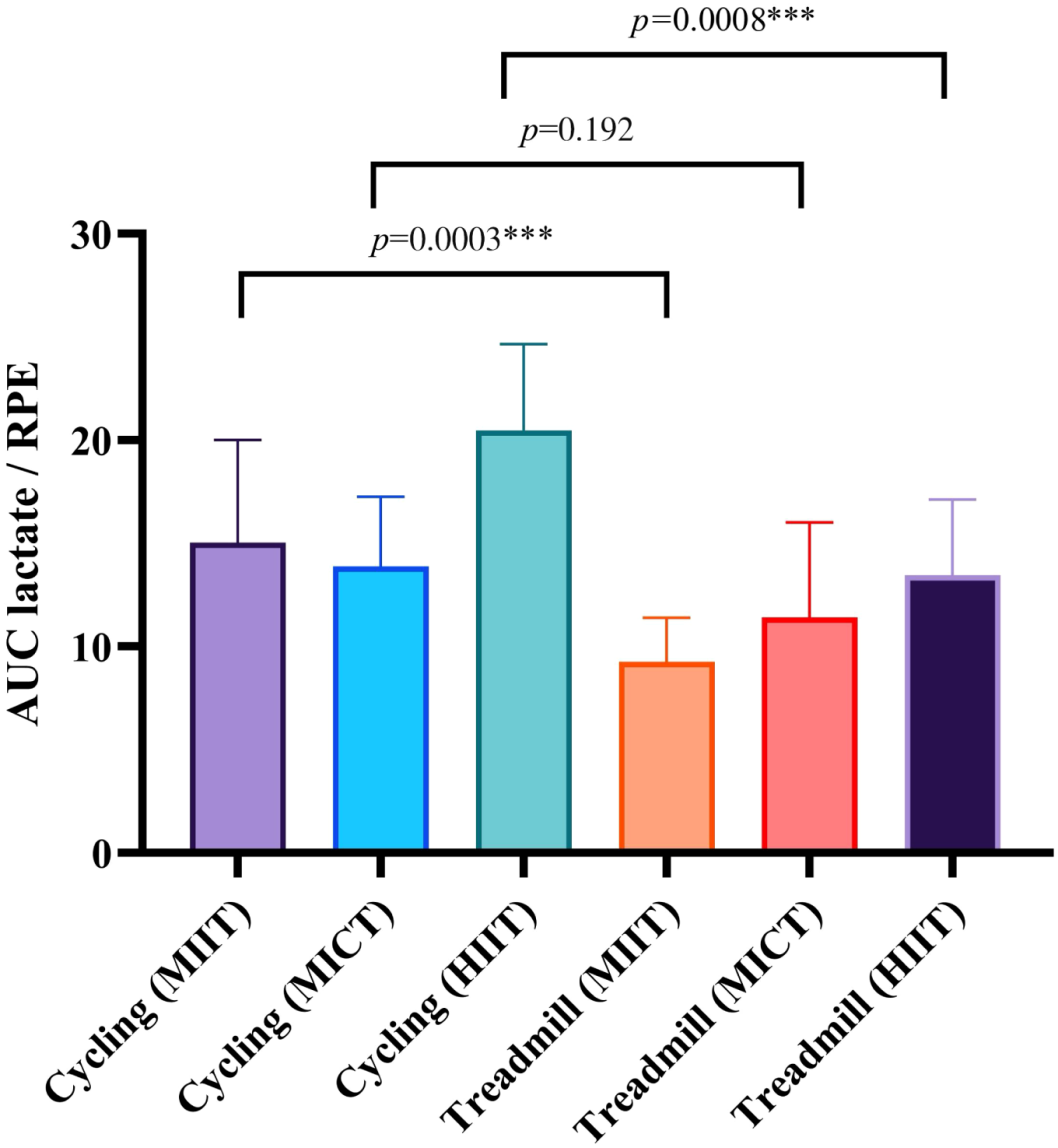
Lactate Production Efficiency (LPE) expressed as the ratio of lactate AUC to mean RPE between modalities cycling and running in three types of protocols(MICT,MIIT,HIIT). Data are presented as mean ± SD (n = 12). LPE represents the systemic lactate exposure generated per unit of subjective fatigue. Cycling protocols (HIIT and MIIT) exhibited significantly higher LPE compared to corresponding running protocols (***p < 0.001), indicating that localized muscle recruitment in cycling induces more potent metabolic stress at equivalent levels of perceived exertion. This metric provides a quantifiable basis for prescribing “high-efficiency” lactate-based interventions.

Analysis using the Lactate Production Efficiency (LPE) metric revealed significant modality-specific differences:

- HIIT: Cycling efficiency (20.48 ± 4.00 mmol·min/L·scale) was 52% higher than running efficiency (13.47 ± 3.51 mmol·min/L·scale) (P = 0.000083,t=6.2196).
- MIIT: Cycling efficiency (15.03 ± 4.76 mmol·min/L·scale) was 62% higher than running efficiency (9.25 ± 2.06 mmol·min/L·scale) (P = 0.000395,t=5.0342).
- MICT: Cycling efficiency (13.87 ± 3.23 mmol·min/L·scale) was 21% higher than running efficiency (11.41 ± 4.41 mmol·min/L·scale) (P = 0.192,t=1.8145; **Figure 5**).

### Significantly higher lactate accumulation in males during running, with sex differences amplifying at higher intensities

**Figure 6:**
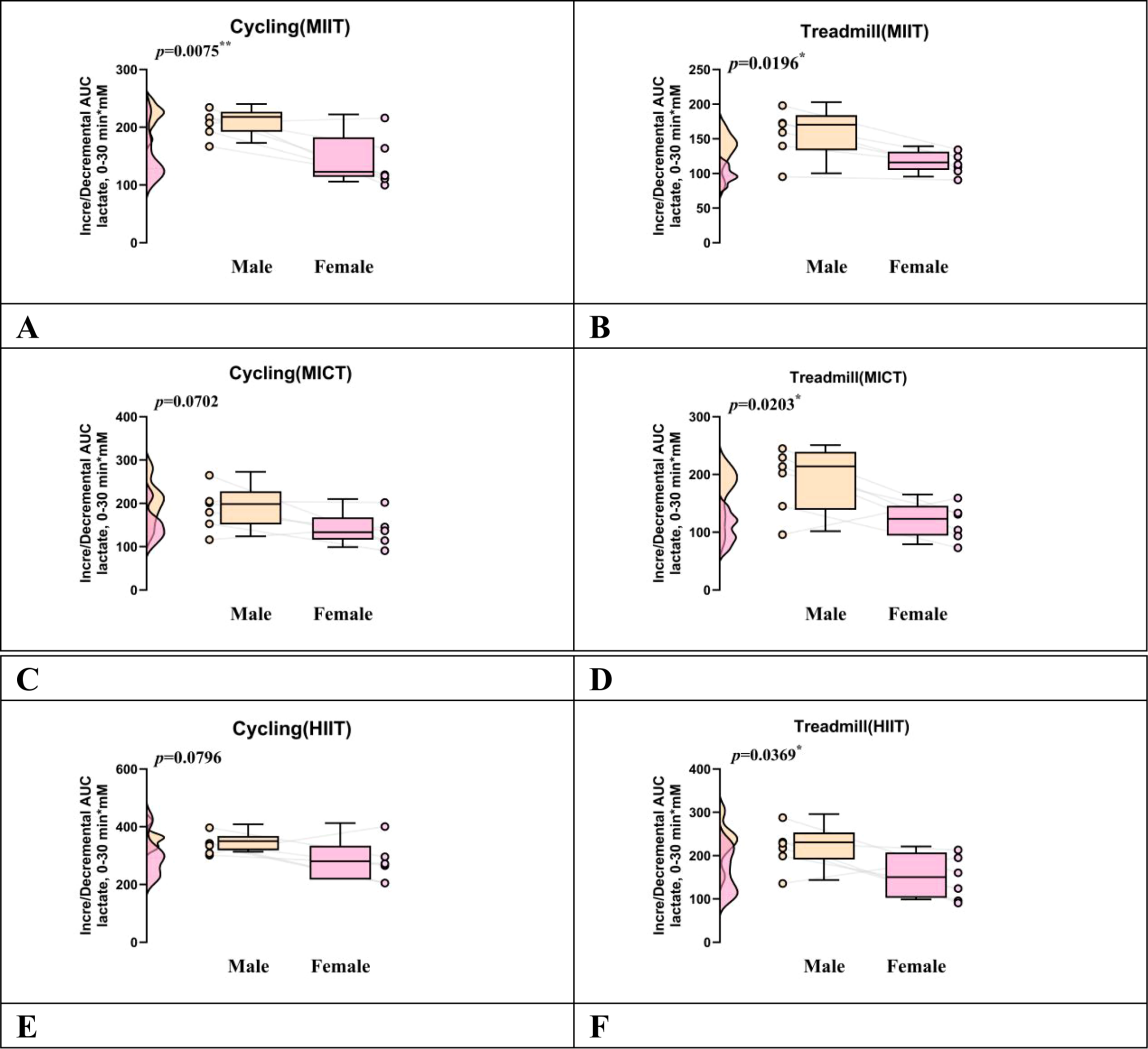
Sex-based differences in blood lactate concentrations across multiple exercise modalities and intensities. Data are presented as mean ± SD (n=12, 6 males and 6 females). **(A, B)** Blood lactate kinetics during MIIT for cycling and running, respectively. **(C, D)** Lactate responses during MICT. **(E, F)** Lactate responses during HIIT. Males generally exhibited higher absolute lactate accumulation compared to females, particularly during running protocols, reflecting potential sex-specific metabolic adaptations to whole-body vs. localized exercise.AUC was measured in mmol/L·min. Statistical significance between sexes at specific time points is indicated by asterisks (*p< 0.05,**p < 0.01).

A sex-stratified analysis revealed significant differences in lactate AUC specifically during running (**Figure 6**):

- During MIIT, MICT, and HIIT running protocols, male participants exhibited significantly higher lactate AUC compared to females:36% higher in MIIT(Male:132.67 ± 25.84 mmol·min/L vs. Female:97.71 ± 11.23 mmol·min/L;P=0.0196,t=2.774), 57% higher in MICT(Male:166.96 ± 43.20 mmol·min/L vs. Female: 106.21 ± 23.79 mmol·min/L;P=0.203,t=2.754), and 43% higher in HIIT (Male:232.33 ± 45.21mmol·min/L vs. Female:162.25 ± 46.83 mmol·min/L;P=0.0369,t=2.407).
- For cycling ergometry, a significant sex difference favoring higher lactate AUC in males was observed only during MIIT (Male: 217.33 ± 21.36 mmol·min/L vs. Female: 149.04 ± 40.48 mmol·min/L;P=0.0075,t=3.337). The sex difference during MICT cycling(Male:202.25 ± 46.27mmol·min/L vs. Female: 149.54 ± 35.22 mmol·min/L; P = 0.0075,t=3.337)and HIIT cycling(Male: 361.04 ± 30.73 mmol·min/L vs. Female: 297.63 ± 65.85 mmol·min/L; P = 0.0075,t=3.337) did not reach statistical significance.

## Discussion

### Mechanistic differences in lactate accumulation: muscle recruitment and metabolic steady states

Our time-course analysis revealed that traditional discrete lactate measurements often mask overall metabolic differences due to temporal fluctuations, particularly in interval training modalities. By utilizing AUC, we mathematically integrated these fluctuations, transforming complex, overlapping kinetic curves into a single, highly sensitive metric of total lactate exposure. This approach effectively resolves the snapshot bias and provides a more robust basis for evaluating cumulative metabolic stress.

The present study demonstrated that cycling ergometry elicits significantly greater lactate accumulation than running at matched target heart rates (**Figure 3**). This divergence is fundamentally driven by the synergistic effects of orthostatic cardiovascular dynamics and distinct muscle recruitment patterns, which together dictate the balance between lactate production and clearance. First, matching intensity via heart rate introduces an orthostatic bias: the fully upright posture of running challenges venous return, eliciting a compensatory increase in heart rate(Truijen, J. et al., 2010). Consequently, achieving the same target heart rate during seated cycling requires a higher absolute mechanical and metabolic workload. Second, this elevated workload in cycling is concentrated on localized lower-limb muscles (primarily the quadriceps and hamstrings). This concentrated effort overloads local mitochondrial oxidative capacity and accelerates the glycolytic pathway, leading to robust lactate accumulation. Conversely, the synergistic, whole-body movement of running not only distributes the workload but also provides a vastly larger “clearance sink”. According to the lactate shuttle theory, the widespread engagement of non-prime-moving muscles (e.g., in the torso and upper limbs) facilitates continuous systemic lactate consumption via mitochondrial oxidation. Coupled with a stronger skeletal muscle pump that accelerates lactate transport for hepatic gluconeogenesis, running achieves a highly efficient systemic lactate turnover (Brooks et al., 2021). Together, the higher localized production requirement in cycling and the superior systemic clearance capacity in running explain the divergent cumulative AUCs despite equivalent cardiovascular prescriptions.

Furthermore, the specific intermittent protocol employed in this study (4 min exercise/1 min rest, yielding a 4:1 work-to-rest ratio) highlights the critical role of recovery duration in modulating not only lactate accumulation but also the Rating of Perceived Exertion (RPE). Previous research indicates that during high-intensity interval training, longer work bouts coupled with proportionally short recoveries (e.g., 60 seconds) often fail to provide sufficient time for lactate removal, thereby accelerating fatigue development and significantly elevating RPE(Myrkos, A.et al., 2022). In contrast, our data reveal that at moderate intensities, this 4:1 ratio did not significantly alter the total lactate load or subjective fatigue perception compared to continuous exercise. This phenomenon suggests that 1 minute of active recovery is physiologically sufficient to clear the lactate generated during a 4-minute moderate-intensity bout. Consequently, this specific work-to-rest ratio facilitates a dynamic equilibrium—matching lactate production during exercise bouts with mitochondrial oxidation during recovery periods—resulting in comparable overall AUC values (**Figure 4**) without exacerbating subjective strain. Notably, our results reveal a profound decoupling of physiological stress from subjective perception: at equivalent RPE levels, intermittent cycling induced a substantially stronger lactate response, whereas intermittent running exhibited the lowest lactate production efficiency. These findings provide a crucial quantifiable basis for “Lactate Production Efficiency” (LPE), emphasizing that manipulating the work-to-rest ratio is a highly effective strategy for the precise titration of lactate exposure and the optimization of customized exercise prescriptions (**Figure 5**).

### Decoupling of subjective fatigue and lactate production

In the present study, while the lactate AUC was significantly higher during cycling compared to running, no significant differences in RPE were observed between the two modalities at matched intensities(**Figure 4C**). This highlights a distinct decoupling phenomenon between subjective fatigue and actual physiological lactate accumulation. This decoupling occurs because RPE represents an integrated, holistic assessment of exercise stress rather than a direct, isolated reflection of blood lactate levels. The genesis of RPE involves the integration of multiple systemic inputs, primarily cardiovascular strain, respiratory effort, and localized muscle feedback. For instance, our data demonstrate that RPE scales consistently with cardiovascular load across different intensities; the significantly higher RPE observed during HIIT compared to MIIT corresponds directly to the higher heart rate zones (80-89% vs. 40-59% HRR, **Table 2**). Consequently, while localized lactate accumulation contributes to peripheral metabolic stress, it does not exclusively dictate the systemic sensation of fatigue. Because RPE is heavily weighted by systemic cardiovascular feedback, it may fail to capture the specific localized metabolic stress inherent to localized modalities like cycling. Therefore, prescribing exercise intensity based solely on subjective metrics like RPE can result in unintended and potentially excessive lactate exposures.

This study introduces, for the first time, the novel metric “Lactate Production Efficiency per Unit RPE” (LPE, AUC/RPE), revealing the superior efficiency of cycling in generating lactate for a given perception of effort. Exercise prescription design must therefore consider the dynamic relationship between RPE and lactate response. For example, cycling can elicit higher lactate exposure at equivalent RPE levels, making it suitable for populations requiring highly efficient lactate elevation (e.g., for metabolic or cognitive benefits). Conversely, the “low RPE - low lactate” characteristic of running may be more appropriate for scenarios where fatigue perception needs to be carefully managed, such as in elderly individuals or rehabilitation patients.

### Exercise-specific nature of sex differences

The significantly higher lactate accumulation observed in males compared to females during running aligns with previous studies reporting greater lactate production in males during high-intensity exercise when relative intensity is controlled (Mochizuki et al., 2020). However, a significant sex difference in cycling was uniquely observed during MIIT, a pattern that may be rooted in the interplay between exercise intensity and inherent muscle fiber composition. As reviewed by Haizlip et al. (2015), females typically possess a higher proportion of type I (slow-twitch, oxidative) muscle fibers and exhibit superior lipid oxidation capacity, whereas males possess a higher proportion of type II (fast-twitch, glycolytic) fibers and greater glycolytic enzyme activity. We hypothesize that these physiological differences are highly intensity-dependent. During HIIT, the extreme metabolic demand likely triggers near-maximal glycolytic activation in both sexes, creating a “ceiling effect” that masks subtle sex-related metabolic nuances. Conversely, during MICT, the low intensity remains well within the aerobic threshold for both groups. However, MIIT likely operates near the aerobic-anaerobic transition zone—a ‘physiological window’ where the superior oxidative capacity of females is most evident. Specifically, key mitochondrial enzymes such as citrate synthase are approximately 15% higher in females, promoting more efficient mitochondrial oxidation of pyruvate and enhanced lactate turnover. Consequently, at equivalent heart rates, females exhibit lower cumulative lactate during MIIT because their muscle profile is better optimized for lactate clearance at threshold intensities. While the localized metabolic stress of cycling often masks sex-specific differences, the specific intensity of MIIT amplifies these underlying biological advantages. As this study did not directly measure muscle fiber types or hormone levels, future research employing molecular biological techniques is warranted to validate this hypothesis.

### Clinical implications

From a translational perspective, the findings of this study have significant clinical implications for the precision titration of exercise prescriptions. Our data suggest that exercise modality and intensity should be strategically matched to the specific therapeutic goals and physiological tolerances of the individual. For populations seeking to leverage the systemic signaling properties of lactate—such as athletes aiming to enhance mitochondrial biogenesis or patients with metabolic syndrome—cycling-based interval protocols (HIIT or MIIT) are recommended, as they provide a potent localized metabolic stimulus. Conversely, for clinical populations requiring minimized systemic metabolic stress, such as cancer patients undergoing treatment or immunocompromised individuals (Bergerot et al., 2024), running-based moderate-intensity protocols appear more suitable. These running-based modalities offer a higher Lactate Production Efficiency (LPE), allowing for the maintenance of cardiovascular benefits while avoiding excessive lactate accumulation. By integrating the LPE framework into clinical practice, practitioners can move beyond “one-size-fits-all” prescriptions toward highly individualized strategies that optimize the balance between metabolic stimulus and physiological strain.

## Conclusion

This study demonstrates that exercise modality significantly modulates the coupling between systemic lactate production and subjective exertion. By introducing the novel Lactate Production Efficiency (LPE) metric, our findings reveal that localized exercise, such as cycling, induces a substantially greater lactate stress response per unit of perceived exertion compared to whole-body exercise like running. This decoupling phenomenon indicates that equivalent levels of subjective fatigue can correspond to markedly different metabolic loads. Specifically, high-intensity interval cycling optimizes this decoupling, offering a “high lactate - manageable fatigue” profile. Ultimately, the LPE framework provides a quantifiable basis for precision exercise prescription, highlighting cycling as a highly efficient modality for interventions requiring potent metabolic stimulation without imposing an excessive perceived burden.

### Study limitations and future directions

- Sample Size and Protocol Limitations: The study involved a relatively small sample size (n=12), and the exercise protocols were laboratory-standardized, potentially limiting generalizability to real-world exercise settings. Future research should expand the sample size and include participants with varying training statuses. While preliminary conclusions are drawn from healthy individuals, factors like age-related muscle decline (sarcopenia) in the elderly or metabolic abnormalities in cancer patients may alter exercise responses, warranting further investigation.
- We acknowledge that using a treadmill-derived VO2max to prescribe cycling intensity is a limitation. However, we used %HRR to monitor intensity during trials, which is a practical and widely used method in cross-modality comparisons.
- Depth of Mechanistic Insight: Direct measurement of metabolic enzyme activity or mitochondrial function in muscle biopsy samples was not performed. Future studies should integrate omics technologies (e.g., proteomics, metabolomics) to further elucidate the molecular mechanisms underlying exercise modality and sex differences.
- Clinical Application Extension: Lactate Production Efficiency per Unit RPE (LPE) holds promise as a potential biomarker for personalized exercise prescription. Its clinical utility, particularly for populations like individuals with diabetes or obesity, requires validation in larger cohorts.

## Data Availability

The original contributions presented in the study are included in the article/supplementary material. Further inquiries can be directed to the corresponding author.
